# Role of high dose rate interstitial brachytherapy in early and locally advanced squamous cell carcinoma of buccal mucosa

**DOI:** 10.1186/2193-1801-3-590

**Published:** 2014-10-09

**Authors:** Parthasarathy Vedasoundaram, Aravind Kumar Prasanna, Reddy KS, Gangothri Selvarajan, Mourougan Sinnatamby, Seenisamy Ramapandian, Saravanan Kandasamy

**Affiliations:** Radiation Oncologists, Department of Radiotherapy, Regional Cancer Center, Jawaharlal Institute of Postgraduate Medical Education and Research, JIPMER, Puducherry – 6, Puducherry, India; Medical Physicists, Department of Radiotherapy, Regional Cancer Center, Jawaharlal Institute of Postgraduate Medical Education and Research, Puducherry, India

**Keywords:** High dose rate interstitial brachytherapy, Buccal mucosal cancer, Organ preservation

## Abstract

**Background:**

The study aimed to assess the effect of High Dose Rate (HDR) Interstitial Brachytherapy when used alone or in combination with External Beam Radiotherapy (EBRT), in early and locally advanced squamous cell carcinoma of buccal mucosa.

**Materials and methods:**

Thirty three patients with histologically proven squamous cell carcinoma of the buccal mucosa received high dose rate interstitial brachytherapy either as primary treatment or as a boost from November 2008 to April 2013. Stage I patients received interstitial brachytherapy alone to a dose of 38.50 Gy, 3.5 Gy per fraction, twice daily at six hours apart for 11 fractions. Stage II patients received EBRT to a dose of 50 Gy in 25 fractions of two Gy each followed by brachytherapy boost to 21 Gy, 3.5 Gy per fraction, twice daily at six hours apart for six fractions. Stage III patients received the same radiotherapy schedule (i.e., same EBRT & Brachytherapy schedule) and with addition of Injection Cisplatin 70 mg/m^2^ in three divided doses every three weeks along with EBRT.

**Results:**

Follow up ranged from 12 to 60 months, median follow up was 26 months. Complete response was observed in 28 patients. Five patients had residual disease and were referred for surgical salvage. One patient died of disease progression. Stage I patients had 100% local control, whereas Stage II and Stage III patients had 84.6% and 80% local control respectively.

**Conclusion:**

HDR Interstitial Brachytherapy used either as a primary treatment modality or as a boost in buccal mucosal cancers provides results comparable to that of surgery, with the advantages of organ preservation, better cosmetic and functional outcomes.

## Introduction

Brachytherapy has been used in the management of cancers since the advent of radiotherapy as a treatment modality in Oncology. It has proven its utility in the treatment of localized tumors with its ability to provide high doses to the target volume while sparing the surrounding normal tissues due to its rapid dose fall-off. This has enabled brachytherapy to be an efficient and cost effective form of conformal radiation therapy, now widely utilized as a significant component of the standard treatment modality in specific cancers including that of cervix, prostate, breast, head and neck, etc., (Mazeron et al. 2003).

Surgery has been the primary modality of treatment in early T1 &T2 buccal mucosal cancers. However, with comparatively better cosmetic and functional outcomes, similar local control and survival figures, radiotherapy has become a preferred alternative to surgery and is recommended by the current NCCN guidelines as a standalone treatment option in these cancers. Brachytherapy alone can be used in the treatment of specific T1N0 cancers of buccal mucosa (Ballonoff et al. 2006; Palme et al. 2004; Pernot et al. 1995).

Though Surgery with post-operative radiotherapy has been standard approach for locally advanced head and neck cancers, concurrent chemotherapy with radiotherapy can be an alternative according to recent NCCN guidelines 2014, but the role of brachytherapy boost is yet to be defined. EBRT with HDR brachytherapy boost has been shown to provide better local control rates compared to external beam radiation alone in locally advanced oral cavity cancers (Donath et al. 1995). Also, concurrent chemo radiation with Injection Cisplatin has proven its superiority over radiotherapy alone in locally advanced head and neck cancers (Studer et al. 2007). However, very few studies have utilized concurrent chemo radiation with brachytherapy boost in these situations.

Various forms of brachytherapy applications including interstitial implants, stents, and molds have been used to treat buccal mucosal cancers (Friedrich et al. 1995; Yoden et al. 1999; Ngan et al. 2005). Most of the earlier studies in these cases have been done using Low Dose Rate (LDR) brachytherapy (Conill et al. 2007). In this study HDR brachytherapy with Iridium 192 source, remotely after loaded via flexible catheters implanted interstitially in the tumor was used. The purpose of this study was to investigate the utility and benefit of brachytherapy with or without EBRT as the sole modality of treatment in early buccal mucosal cancers and as a part of concurrent chemo radiation in locally advanced tumors, in Indian patients.

## Materials and methods

The study included 33 patients with histologically proven squamous cell carcinomas of the buccal mucosa who attended the outpatient department of the Regional Cancer Centre in Jawaharlal Institute of Postgraduate Medical Education & Research (JIPMER) Hospital, Puducherry, from November 2008 to April 2013. Of these, 19 were males and 14 females, with ages ranging from 39 to 65 years (Mean – 52.85 years). Only patients with T1-T3/N0-N1 stage cancers and ECOG performance scores with 0,1or 2 were recruited for the study. Table 1 shows a summary of the patient profiles according to age, gender, performance status, habits, tumor stage and growth pattern.

**Table 1:** **Patient’s profile**

Distribution	Number	Percent
Number of patients	33	
Age group (in years)	Range: 39 to 65, Mean-52.85	
Male	19	57.57
Female	14	42.42
**Habits**		
Tobacco chewing	21	63.63
Smoking	18	54.54
Alcohol	11	33.33
Betel nut chewing	21	63.63
None	2	6.06
**Tumor growth characteristics**		
Infiltrative disease	17	51.51
Proliferative disease	13	39.39
Both	3	9.09
**Disease status and treatment**		
T1 Disease	5	15.15
T2 Disease	16	48.48
T3 Disease	12	36.36
N0	28	84.84
N1	5	15.15
Stage 1	5	15.15
Stage II	13	39.4
Stage III	15	45.45
EBRT	13	39.39
CCRT	15	45.45

*Abbreviations*: *EBRT* External beam radiotherapy, *CCRT* Concurrent Chemo Radiotherapy.

Complete staging workup including thorough physical examination, biopsy of the primary site, Fine Needle Aspiration Cytology (FNAC) of clinically identified lymph nodes, Chest X-ray, Contrast Enhanced Computed Tomography (CECT) scan of head and neck if needed, and baseline blood counts and biochemistry were done for all the patients. Anesthetic fitness was obtained for the brachytherapy procedure. All patients were staged using American Joint Committee on Cancer (AJCC) - 7^th^ Edition recommendations and treatments provided accordingly (Sobin et al. 2009).

### Treatment protocol, planning and implementation

Primary brachytherapy alone was considered for treatment in five patients with Stage I disease. They received interstitial brachytherapy at a dose of 3.5 Gy per fraction, two fractions per day at six hours apart for 11 fractions, to a total dose of 3850 cGy. 13 patients with Stage II disease received EBRT and brachytherapy boost, EBRT was delivered to a dose of 50 Gy in 25 fractions of two Gy each, five fractions per week for 5 weeks. Brachytherapy boost dose was 21 Gy given in six fractions of 3.5 Gy each, twice daily at 6 hours apart, given within two to four weeks of EBRT. 15 patients with Stage III disease received the same radiation treatment schedule i.e., EBRT was delivered to a dose of 50 Gy in 25 fractions of two Gy each, five fractions per week for 5 weeks. Brachytherapy boost dose was 21 Gy given in six fractions of 3.5 Gy each, twice daily at 6 hours apart, given within two to four weeks of EBRT with addition of two cycles of Injection Cisplatin at 70 mg/m^2^ in three divided dose, three weeks apart, concurrently with EBRT. Node positive patients received brachytherapy boost for dose of 21Gy in 6 fractions.

For external beam radiation all patients were positioned supine, immobilized with head rest and thermoplastic mask. Simulation was done in Varian Acuity 2-D simulator. Radiation was delivered by conventional 2-D technique with antero-lateral wedged pair fields using 6MV photons, in Clinac600C Linear Accelerator.

After proper evaluation, brachytherapy procedure was done under general anesthesia. Flexible catheters were placed as double plane implant. A non-contrast planning CT scan of the implanted region with one millimeter thick slices was taken on the second day after the procedure using Somatom Spirit (Siemens) CT. The images were then transferred to the treatment planning systems, either Brachy Vision (Varian Medical Systems, Palo Alto, CA) or Oncentra Master Plan (Nucletron, BV). The Clinical Target Volume (CTV) was delineated and the applicators reconstructed. A reference point was inserted at the tip of all the applicators. Dose optimization was done by adjusting dwell times in individual dwell positions to ensure that at least 90 percent of the CTV received the prescribed dose. AAPM TG-43 formalism was used to generate the dose distributions. Cumulative Dose Volume Histogram (DVH) was used to study the dosimetric parameters.

### Follow-up

Patients were followed up every 2 months in the first year, every 3 months in the second year and every 4 months thereafter, for a period of 12 months to 60 months.

### Statistical analysis

Profile of baseline characteristics and stage and treatment based patient distributions are presented as frequencies and percentages. Dose parameters are expressed as means. Subgroup analysis of treatment outcomes were done with Pearson’s Chi square test and Fisher’s exact test. Analysis of dose parameters and toxicities was done using Binary Logistic regression analysis and Spearman’s correlation test. 5% level of significance was used for all statistical tests and a p-value of < 0.05 was considered statistically significant. SPSS software (version 20) was used for the analysis.

### Ethical considerations

Informed consent was obtained from all participants after proper explanation of the treatment procedure in their vernacular language. Ethical clearance was obtained from competent authority.

Ethical committee clearance was obtained vide number SEC/2011/4/101, approved on 30/11/2011.

## Results

A total of 33 patients were recruited for the study from November 2008 to April 2013. The baseline characteristics are summarized in Table 1.

There were five patients (15.15%) with Stage I disease, 13 patients (39.4%) with Stage II and 15 (45.45%) with Stage III disease. Five patients (15.15%) had node positive (N1) disease. 13 patients (39.39%) with Stage II received external beam radiation and brachytherapy boost. 15 patients (45.45%) with Stage III disease received external beam radiation and brachytherapy boost with Injection Cisplatin 70 mg/m^2^ given concurrently with external beam radiation. 31 patients (94%) had one or more of these habits including alcohol intake

**Figure 1:**
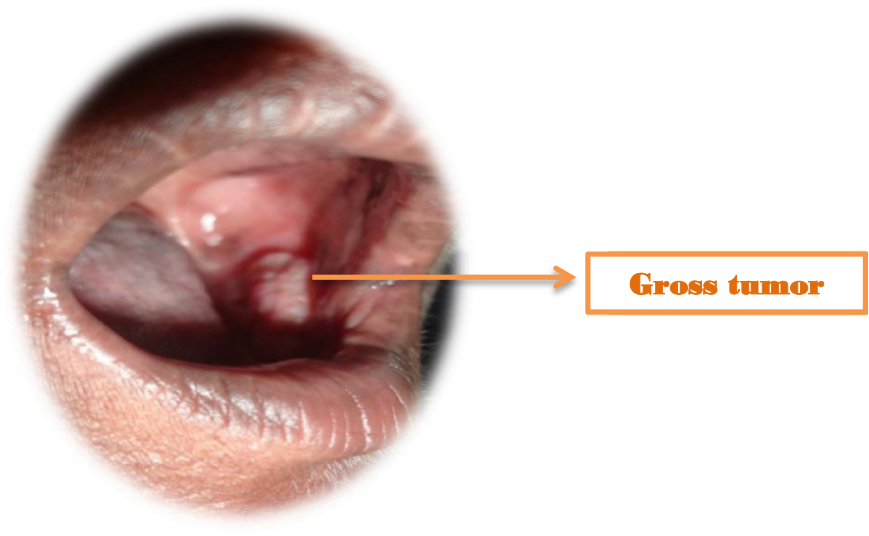
**Pre-treatment gross tumor seen in Left Buccal mucosal carcinoma.**

**Figure 2:**
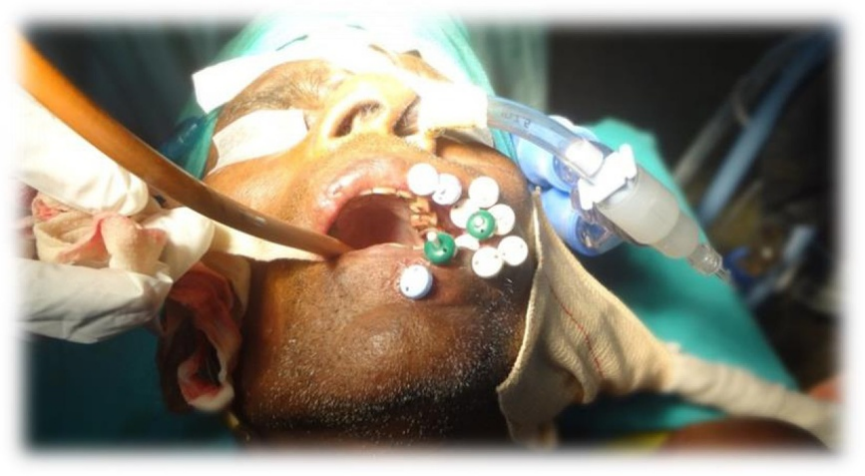
**Interstitial implant procedures done in Left Buccal mucosal Carcinoma.**

**Figure 3:**
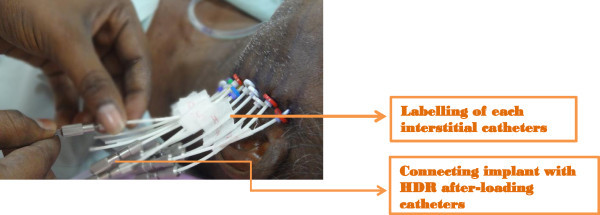
**Interstitial implants connected to HDR after loading catheters.**

**Figure 4:**
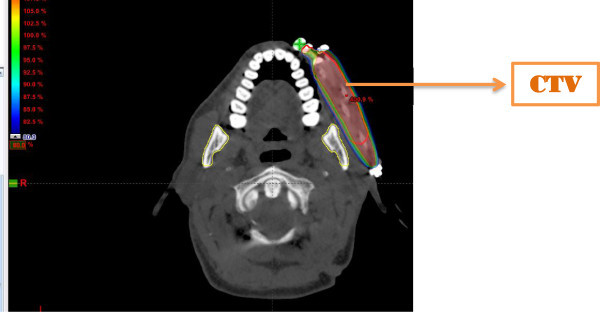
**CTV and Dose colourwash in left Buccal mucosal Carcinoma showing adequate target volume coverage.**

**Figure 5:**
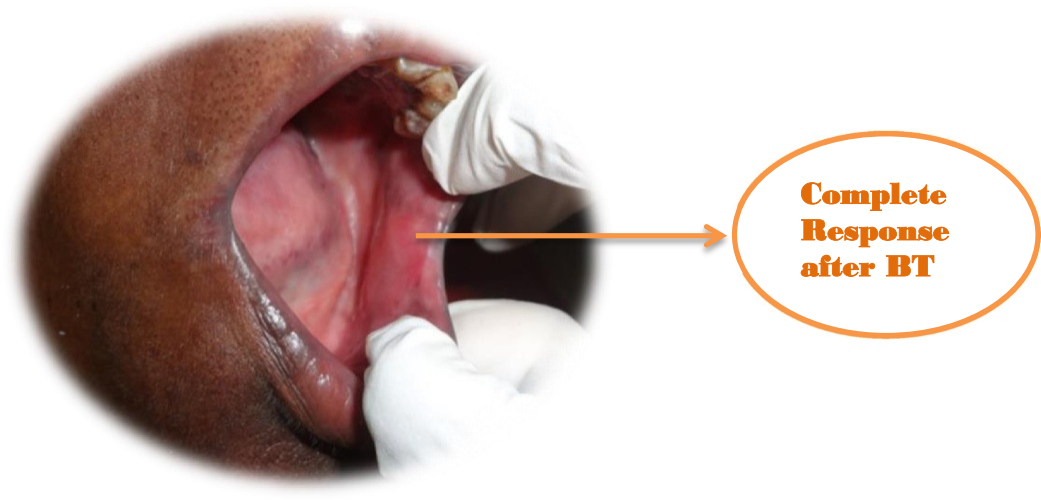
**Complete response seen in left Buccal mucosal carcinoma after completion of treatment on follow up.**

Table 2 shows the overall outcome of the treatment. 28 patients (84.84%) had complete response. Figures 1, 2, 3, 4 and 5 shows the pretreatment evaluation, treatment and post treatment follow-up of one patient; who is disease free till date. Five patients (15.15%) had residual disease. All patients with residual disease were referred to surgical oncology department for salvage surgery. Four patients had good salvage surgery and had no further events during the follow-up period. One patient progressed after surgery and was given palliative chemotherapy with Injection Cisplatin and Paclitaxel. But the disease progressed and ultimately the patient succumbed to the disease.

**Table 2:** **Overall treatment outcome**

Distribution	Number	Percent
Complete response	28	84.84
Residual disease	5	15.15
Expired due to cancer progression	1	3.03
Expired (Total)	1	3.03

Table 3 shows the local response rates in relation to the disease Stage. 5/5 patients (100%) with Stage I disease had complete response. Rates of complete responses were lower with Stage II and Stage III diseases but the differences among the groups were not statistically significant. Sub group analysis showed no statistically significant difference in treatment outcomes with respect to gender, disease growth characteristics or patient’s habits like smoking, tobacco chewing, betel chewing, etc.,.

**Table 3:** **Comparison of treatment outcome in relation to stage**

Outcome	Stage I		Stage II		Stage III		Total	p value
	Number	%	Number	%	Number	%		
Complete response	5	100	11	84.6	12	80	28	0.6
Residual disease	0	0	2	15.4	3	20	5	
Total	5	100	13	100	15	100	33	
Pearson's Chi square								1.2

Table 4 displays the means of various Brachytherapy indices. Coverage Index (CI), Dose Homogeneity Index (DHI), Dose-Non uniformity Ratio (DNR), Conformal Index (COIN), External Volume Index (EVI) were calculated to know about the CTV coverage of implant and to assess the effect of these indices on local control and toxicities related to brachytherapy boost. The above indices did not show any statistically significant differences in local control based on Logistic regression analysis and Spearman’s correlation.

**Table 4:** **Brachytherapy indices**

Indices	CR		R	
	Mean	SD	Mean	SD
**CI**	0.874851	0.053707	0.750564	0.014628
**DHI**	0.682676	0.090335	0.696503	0.04411
**DNR**	0.317324	0.090335	0.303497	0.04411
**EVI**	0.032005	0.013143	0.09421	0.052554
**COIN**	0.844155	0.056749	0.668754	0.029791

*Abbreviations*: *CR* complete response, *R* residual disease, *CI* Coverage index,

*DHI* Dose Homogeneity index, *COIN* conformal index, *DNR* Dose Non uniformity Ratio,

EVI-external volume index, SD- Standard deviation.

Brachytherapy indices like Coverage Index (CI), Dose Homogeneity Index (DHI), Dose-Non uniformity Ratio (DNR), Conformal Index (COIN) and External Volume Index (EVI) were calculated in this study and compared with the reference dose (Feuvret et al. 2006; Kehwar et al. 2008). These indices rely on 3D image-based approach and volume based optimization. In this study Clinical Target Volumes were contoured and dose was prescribed to the isodose covering the target (Mazeron et al. 2009).

Clinical Target Volume covered by 200%, 150%, 100% and 90% of isodoses did not show any statistically significant differences in local control based on Logistic regression analysis and Spearman’s correlation.

Tables 5 shows the various acute and late toxicities observed in these patients. Toxicities where graded using Radiation Therapy Oncology Group (RTOG) criteria (Cox et al. 1995). Acute mucositis and skin reactions were the most common acute radiation toxicities which occurred in approximately 80% of the patients. There was one grade 3 skin reaction which required a brief treatment interruption. Acute nausea and vomiting were observed in about 40% and 24% of patients respectively, commonly in patients on concurrent chemo radiation. Only one patient had soft tissue necrosis.

**Table 5:** **Toxicities observed during ebrt/ccrt and on follow up**

Acute toxicities		N	%
Acute skin reaction	G0	5	15.15
	G1	17	51.51
	G2	10	30.3
	G3	1	3.03
Acute mucositis	G0	5	15.15
	G1	17	51.51
	G2	11	33.33
	G2	2	6.06
Acute nausea	NO	20	60.6
	YES	13	39.39
Acute vomiting	G0	25	75.75
	G1	7	21.21
	G2	1	3.03
G2 Neutropenia	NO	27	81.81
	YES	6	18.18
**Late toxicity**			
Soft tissue necrosis		1	3.03
Death		1	3.03

*Abbreviations*: *N* number, *%* percentage, *G* grade.

## Discussion

With invention of newer technologies and highly conformal dose delivery, modern radiotherapy has set a high standard for the management of oral cavity cancers. The ability to preserve normal anatomy and provide better cosmetic and functional outcomes has made radiotherapy an effective alternative to surgery, which has been the gold standard for management of both early and locally advanced oral cavity cancers from time immemorial (Matsui et al. 2007). Three Dimensional Conformal radiotherapy (3D CRT) and Intensity Modulated Radiotherapy (IMRT) have made possible higher dose delivery with curative intent to the tumor, with acceptably lower doses to normal organs and critical structures in its neighborhood (Studer et al. 2007). However, higher costs and complexity in planning and treatment delivery have precluded their widespread adoption, especially in third world nations, where cost effectiveness and ease of implementation are the need of the hour (Nijdam et al. 2008).

Brachytherapy has proven itself indispensable in the management of specific cancers like cancer cervix and oral cavity cancers over the decades, as a primary modality or as a boost. Its lower cost and simplicity, coupled with its ability to provide high localized dose with rapid dose fall off has made it an excellent tool to provide conformal therapy in these cancers, with minimal side effects compared to EBRT. A study by Sresty et al. ( 2010) showed that interstitial brachytherapy conferred more dose homogeneity when compared with IMRT and lesser dose to critical structures. Also planning time was much less for most cases. It concluded that interstitial brachytherapy was an ideal option for high dose delivery exclusively to the primary tumor volume, while limiting the risk of severe xerostomia or trismus.

From the present study we can infer that HDR Interstitial brachytherapy alone is a viable option for management of small localized buccal mucosal cancers. Five patients with T1N0 tumors were treated with HDR brachytherapy alone to a dose of 38.50 Gy and all of them had complete response at the end of treatment and continued progression free throughout the follow up period.

The extent of the primary lesion and the presence of nodal disease were found to have an impact on the local control. Patients with Stage I disease had 100% local control whereas Stage II and III patients had 84.6% and 80% local control respectively. This outcome is similar to other published results (Hiratsuka et al. 1997; Shear et al. 1976). Survival following primary surgery for oral cancer showed similar statistics (Simon et al. 2009).

Most of the published literatures in interstitial brachytherapy for oral cavity cancers were based on LDR and manual after loading techniques (Conill et al. 2007). With the recent advances in 3D image based techniques, the possibility of accurate delineation of the target volumes and optimization of dose distributions have made HDR brachytherapy much safer to patients and disease control better (Harrison 1997; Strnad 2004).

According to the American Brachytherapy Society recommendations Coverage Index of 1, Dose Homogeneity Index of more than 0.75 and External Volume Index of zero (EVI = 0) should be achieved with more than 90% of dose delivered to >90% target volume. Nag et al. ( 2001) stated that Conformal index (COIN) should be one (1) in order to achieve better quality of tumor irradiation and normal tissue sparing by interstitial brachytherapy.

In this study group the mean Coverage Index was 0.87 (ideal 1), Dose Homogeneity Index 0.7 (ideal 0.75), Dose-Non uniformity Ratio 0.3 (ideal 0.1), Conformal Index 0.84 (ideal 1) and External Volume Index 0.03 (ideal 0.05). All the parameters were well within the recommended range.

Patients with near ideal Coverage Index and Conformity Index showed a trend to complete response compared to those with residual disease. However Logistic Regression Analysis showed that these trends did not graduate to statistical significance. There were no observable differences with other indices with respect to the disease response. Still, we can observe from this study that good brachytherapy technique definitely plays a role in obtaining complete response.

Tobacco chewing, smoking and betel nut chewing are known to be significant factors in the etiopathogenesis of oral cavity cancers (Llewellyn et al. 2001). In this study sample, 31 patients (94%) had one or more of these habits including alcohol intake. However in subgroup analysis, neither of these habits individually correlated with the disease outcome. Similarly, no correlation to disease outcome was observed with the histological grade or the morphological growth pattern of the tumor.

The treatment was well tolerated by most patients. The most common side effects observed during radiation were acute mucositis and skin reactions (80% of patients). 50% had grade I toxicities and 30% had grade II. All of them were managed conservatively with antiseptic oral rinses and zinc containing multivitamins which improved the symptoms. One patient had grade III skin reaction during external beam radiation and radiation had to be briefly interrupted to allow healing (Emami et al. 1991). Patients with locally advanced diseases were given concurrent Cisplatin chemotherapy with external beam radiation followed by brachytherapy boost (15 patients, 45%) after two weeks of EBRT. Acute nausea and vomiting were observed in 40% and 24% of these patients and were managed effectively with antiemetics.

Six patients (18%) developed grade II neutropenia following chemotherapy and were managed with regular monitoring of blood counts and antibiotics whenever necessary. Few patients had treatment interruptions and delay in brachytherapy due to these acute toxicities (8 patients). However, there was no statistically significant effect on the local control with these delays.

8 month after completion of treatment one patient developed soft tissue necrosis. She was managed conservatively with analgesics and antibiotics. She was symptom free after six months of close follow up period (Emami et al. 1991).

## Conclusion

Surgery with or without adjuvant radiotherapy has been the standard of treatment for buccal mucosal cancers. Most of the buccal mucosal cases are operated by Surgeons. Number of early buccal mucosal cases reported to Radiation Oncology department is small. We can say from the study that EBRT with brachytherapy boost can produce survival rate and local control comparable to surgery alone (Simon et al. 2009). With lower morbidity, better cosmetic and functional outcomes and comparable local control and survival rates, radiotherapy has slight advantage over surgery in early and locally advanced buccal mucosal lesions. Good brachytherapy technique and meticulous planning are essential to ensure adequate dose coverage of the tumor. Well planned multiple plane implants with adequate equally spaced flexible catheters will give more room for uniform dose delivery and translate to near ideal brachytherapy indices and better treatment outcomes.
